# Semaglutide-Mediated Remodeling of Adipose Tissue in Type 2 Diabetes: Molecular Mechanisms Beyond Glycemic Control

**DOI:** 10.3390/ijms27031186

**Published:** 2026-01-24

**Authors:** Tatjana Ábel, Éva Csobod Csajbókné

**Affiliations:** Department of Dietetics and Nutritional Sciences, Faculty of Health Sciences, Semmelweis University, 1088 Budapest, Hungary; csajbokne.csobod.eva@semmelweis.hu

**Keywords:** adipose tissue remodeling, beige adipocytes, extracellular matrix remodeling, GLP-1 receptor agonist, immunometabolism, mitochondrial biogenesis, semaglutide, type 2 diabetes mellitus, visceral adiposity

## Abstract

Type 2 diabetes mellitus (T2DM) is characterized not only by chronic hyperglycemia but also by profound adipose tissue dysfunction, including impaired lipid handling, low-grade inflammation, mitochondrial dysfunction, and extracellular matrix (ECM) remodeling. These adipose tissue alterations play a central role in the development of systemic insulin resistance, ectopic lipid accumulation, and cardiometabolic complications. Glucagon-like peptide-1 receptor agonists (GLP-1RAs), particularly semaglutide, have emerged as highly effective therapies for T2DM and obesity. While their glucose-lowering and appetite-suppressive effects are well established, accumulating evidence indicates that semaglutide exerts pleiotropic metabolic actions that extend beyond glycemic control, with adipose tissue representing a key target organ. This review synthesizes current preclinical and clinical evidence on the molecular and cellular mechanisms through which semaglutide modulates adipose tissue biology in T2DM. We discuss depot-specific effects on visceral and subcutaneous adipose tissue, regulation of adipocyte lipid metabolism and lipolysis, enhancement of mitochondrial biogenesis and oxidative capacity, induction of beige adipocyte programming, modulation of adipokine and cytokine secretion, immunometabolic remodeling, and attenuation of adipose tissue fibrosis and ECM stiffness. Collectively, available data indicate that semaglutide promotes a functional shift in adipose tissue from a pro-inflammatory, lipid-storing phenotype toward a more oxidative, insulin-sensitive, and metabolically flexible state. These adipose-centered adaptations likely contribute to improvements in systemic insulin sensitivity, reduction in ectopic fat deposition, and attenuation of cardiometabolic risk observed in patients with T2DM. Despite compelling mechanistic insights, much of the current evidence derives from animal models or in vitro systems. Human adipose tissue-focused studies integrating molecular profiling, advanced imaging, and longitudinal clinical data are therefore needed to fully elucidate the extra-glycemic actions of semaglutide and to translate these findings into adipose-targeted therapeutic strategies.

## 1. Introduction

Type 2 diabetes mellitus (T2DM) represents a growing global health burden characterized by chronic hyperglycemia, insulin resistance, and progressive pancreatic β-cell dysfunction [1]. Beyond impaired glucose homeostasis, obesity is strongly associated with ectopic lipid accumulation, chronic low-grade inflammation, and T2DM, which together accelerate cardiometabolic morbidity and mortality [2]. Over the past decade, adipose tissue has shifted from being viewed primarily as a passive energy reservoir to being recognized as a dynamic endocrine and immunomodulatory organ [3]. Through the secretion of adipokines, cytokines, lipokines, and extracellular vesicles, adipose tissue exerts broad control over systemic insulin sensitivity, inflammatory tone, and metabolic adaptation [3].

In T2DM with obesity, adipose tissue becomes dysfunctional—characterized by adipocyte hypertrophy, altered extracellular matrix (ECM) remodeling, immune cell infiltration, and impaired mitochondrial function—thereby promoting insulin resistance and systemic metabolic deterioration [1]. This dysfunctional state contributes to increased free fatty acid flux, ectopic lipid deposition, and a feed-forward cycle of inflammation and metabolic stress [2]. Consequently, adipose tissue is increasingly recognized as a central driver of T2DM pathophysiology and a therapeutically relevant target organ [1,2].

Glucagon-like peptide-1 receptor agonists (GLP-1RAs), particularly semaglutide, have emerged as potent therapeutic agents for both T2DM and obesity. Although their primary clinical benefits include glucose-dependent insulin secretion and appetite regulation, growing evidence indicates a wider spectrum of metabolic effects that extend beyond glycemic control [4,5,6,7]. Semaglutide has demonstrated sustained weight reduction, improvements in dyslipidemia, reduction in cardiovascular risk, and clinically meaningful modulation of adipose tissue-related metabolic risk [4,5,6,7]. Notably, clinical studies indicate that semaglutide preferentially reduces visceral adipose tissue (VAT), improves body composition, and attenuates adipose-driven inflammatory signaling [8,9].

Mechanistic investigations further suggest that semaglutide influences adipocyte function and adipose tissue remodeling through multiple, potentially convergent pathways. These include enhancement of beige/brown adipocyte gene programs, stimulation of mitochondrial biogenesis, and activation of AMPK–SIRT1–PGC-1α signaling cascades [10,11]. Preclinical studies also report attenuation of oxidative stress, suppression of pro-inflammatory macrophage polarization, and partial normalization of adipokine secretion patterns [12,13]. Together, these pleiotropic actions support the concept that adipose tissue represents an important site through which semaglutide may contribute to systemic metabolic improvement, complementing classical incretin-mediated mechanisms [14].

Taken together, a comprehensive understanding of how semaglutide modulates adipose tissue at molecular, cellular, and depot-specific levels is essential for defining its full therapeutic potential in T2DM. Accordingly, the objective of this review is to provide an integrated and mechanistic overview of the emerging evidence on semaglutide-induced adipose tissue remodeling beyond glycemic control.

Specifically, this review aims to achieve the following:

- Summarize the pathophysiological features of adipose tissue dysfunction in T2DM that underpin insulin resistance and cardiometabolic risk;
- Examine depot-specific effects of semaglutide on visceral, subcutaneous, and epicardial adipose tissue;
- Delineate the molecular mechanisms by which semaglutide regulates adipocyte lipid metabolism, lipolysis, and ectopic fat deposition;
- Discuss mitochondrial adaptations and the induction of beige/brown adipocyte programs associated with semaglutide treatment;
- Evaluate the immunometabolic effects of semaglutide on adipokine secretion and inflammatory signaling.

## 2. Adipose Tissue in Type 2 Diabetes: Pathophysiology

### 2.1. Adipocyte Hypertrophy, Inflammation, and Insulin Resistance

**Figure 1:**
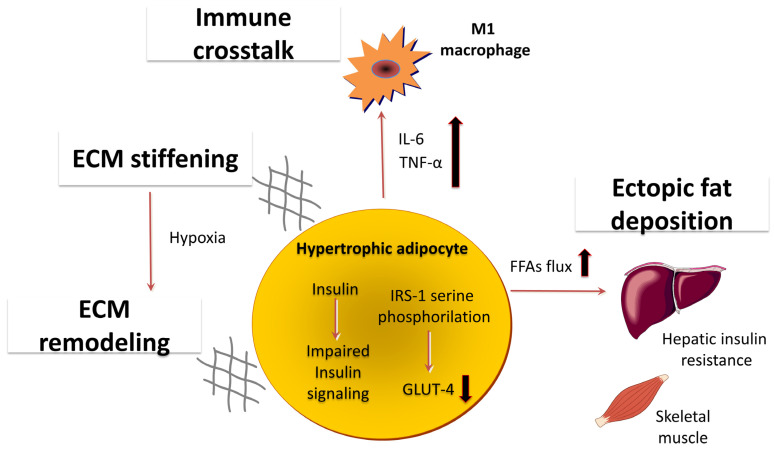
Pathophysiological features of adipose tissue dysfunction in type 2 diabetes mellitus. In T2DM, adipose tissue expansion is dominated by adipocyte hypertrophy, leading to local hypoxia, extracellular matrix (ECM) stiffening and remodeling, and impaired insulin signaling. Hypertrophic adipocytes exhibit increased free fatty acids (FFAs) release and altered adipokine secretion, promoting immune crosstalk with pro-inflammatory M1 macrophages and the release of cytokines such as IL-6 and TNF-α. These processes disrupt insulin receptor signaling through inhibitory serine phosphorylation of IRS-1 and reduced GLUT-4-mediated glucose uptake. Enhanced FFAs flux, particularly from visceral adipose tissue, contributes to ectopic fat deposition and hepatic insulin resistance, thereby reinforcing systemic metabolic dysfunction.

In the context of obesity and T2DM, adipose tissue expansion is often dominated by adipocyte hypertrophy rather than healthy hyperplasia, leading to reduced lipid storage capacity and increased lipotoxic exposure of peripheral organs such as the liver and skeletal muscle (Figure 1) [15].

Hypertrophic adipocytes experience bioenergetic and mechanical stress and exhibit an altered secretory profile, characterized by elevated release of pro-inflammatory cytokines (e.g., interleukin-6 (IL-6), tumor necrosis factor-α (TNF-α)) and chemokines such as C-C motif chemokine ligand 2 (CCL2), thereby promoting immune cell recruitment to the adipose microenvironment [16].

Within inflamed adipose tissue, key insulin receptor signaling intermediates are disrupted: insulin receptor substrate 1 (IRS-1) becomes serine-phosphorylated, and glucose transporter type-4 (GLUT4) expression and translocation are reduced, collectively impairing insulin-mediated glucose uptake and potentiating systemic insulin resistance [17]. Concomitantly, local hypoxia, oxidative stress, and maladaptive ECM remodeling further exacerbate adipocyte dysfunction, underscoring the multifactorial nature of adipose tissue-driven metabolic derangement in T2DM [18].

### 2.2. Lipotoxicity and Ectopic Fat Deposition

When adipose tissue’s capacity to safely store excess nutrients is exceeded or functionally impaired, free fatty acids (FFAs) flux increases, resulting in ectopic lipid deposition in non-adipose organs such as the liver, pancreas, and skeletal muscle, thereby exacerbating insulin resistance and β-cell stress [19]. This process reflects impaired adipose tissue buffering rather than excess adiposity per se.

VAT particularly contributes to this phenomenon owing to its high lipolytic activity and direct portal drainage to the liver; accordingly, visceral fat accumulation is more strongly associated with metabolic dysfunction than subcutaneous depots [20]. Importantly, impaired adipogenesis and limited adipose tissue expandability have emerged as critical determinants of lipid spillover, potentially representing early indicators of metabolic disease risk [21].

### 2.3. Role of Adipokines and Immune Crosstalk

In health, adipose tissue secretes beneficial adipokines (e.g., adiponectin) that regulate energy balance, insulin sensitivity, and inflammation. In the context of T2DM, the adipokine secretory profile becomes dysregulated, with reduced levels of insulin-sensitizing adipokines and increased secretion of pro-inflammatory adipokines and cytokines, thereby disrupting metabolic homeostasis [22]. Simultaneously, adipose tissue undergoes marked immunological remodeling, characterized by increased infiltration of M1-polarized macrophages, pro-inflammatory T-cell subsets, and other stromal–immune populations [23]. This reciprocal crosstalk between adipocytes, immune cells, and stromal components establishes a feed-forward loop of inflammation, adipose tissue dysfunction, and insulin resistance, positioning the adipose immune microenvironment as a critical axis in the pathogenesis of T2DM and a promising therapeutic target.

## 3. Semaglutide: Pharmacology and Mechanisms of Action

### 3.1. GLP-1 Receptor Signaling in Metabolic Tissues

**Figure 2:**
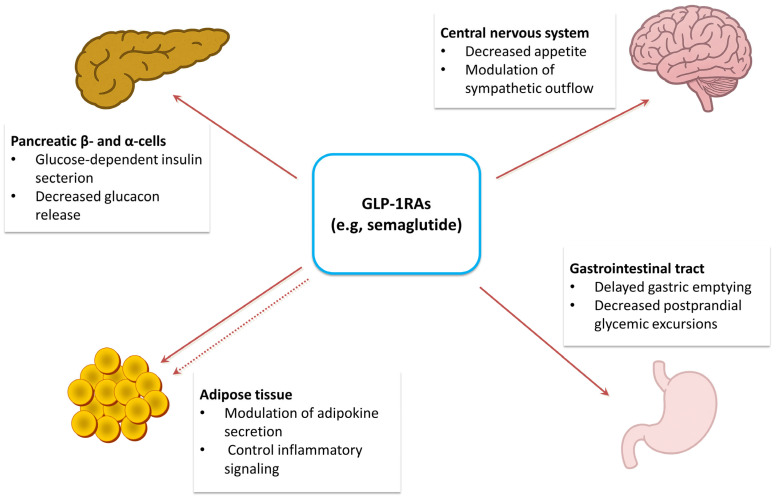
Mechanisms of action of GLP-1 receptor agonists across organ systems.

Semaglutide is a long-acting glucagon-like peptide-1 receptor agonist (GLP-1RA) that binds to GLP-1 receptors expressed on pancreatic β-cells and within the central nervous system (CNS). Importantly, emerging evidence suggests that GLP-1 receptors are also functionally relevant in adipose tissue-associated cell populations, positioning adipose tissue as a potential indirect and direct target of GLP-1RAs’ actions (Figure 2) [24].

Upon activation of the GLP-1 receptor (GLP-1R), several downstream events are triggered, including glucose-dependent insulin secretion, suppression of glucagon release, delayed gastric emptying, and reinforcement of satiety signals [25]. Beyond these canonical pancreatic and gastrointestinal pathways, GLP-1R signaling exerts pleiotropic metabolic effects via central (hypothalamic–sympathetic) and peripheral mechanisms, influencing lipolysis, lipid oxidation, adipokine secretion, and adipose tissue remodeling [26].

GLP-1 receptor agonists (GLP-1RAs), such as semaglutide, exert effects on multiple organ systems, including the central nervous system, pancreas, adipose tissue, and gastrointestinal tract. These effects include appetite suppression, enhanced glucose-dependent insulin secretion, reduced glucagon release, delayed gastric emptying, and modulation of inflammatory and adipokine signaling.

### 3.2. Pharmacokinetics and Dosing

Semaglutide’s pharmacokinetic profile supports its once-weekly subcutaneous administration. Semaglutide is 94% homologous to endogenous GLP-1 and has a prolonged elimination half-life of approximately 168 h in humans.

The longer elimination half-life allows for sustained plasma concentrations and continuous binding to GLP-1R beyond the acute effects following a meal [27,28]. This extended half-life is achieved through albumin-binding modifications and structural acylation, which reduce dipeptidyl peptidase-4 (DPP-4)-mediated degradation and renal clearance [29].

These pharmacokinetic properties are particularly relevant when considering semaglutide’s effects on adipose tissue, as sustained receptor activation may facilitate long-term remodeling of metabolic and inflammatory pathways rather than transient glycemic modulation alone.

Semaglutide has two main clinical indications—T2DM and obesity—with distinct dose-escalation strategies and target maintenance doses. In T2DM, treatment is initiated at 0.25 mg once weekly for 4 weeks, followed by escalation to 0.5 mg once weekly, with further up-titration to 1.0 mg once weekly depending on glycemic targets and tolerability. In contrast, in obesity management, semaglutide is typically titrated stepwise to a higher maintenance dose of 2.4 mg once weekly, using gradual escalation to improve gastrointestinal tolerability. These indication-specific regimens are relevant for mechanistic interpretation, because higher dose exposure and differing baseline metabolic states in obesity-focused cohorts may yield larger weight-loss responses and distinct endpoint profiles compared with T2DM trials, where glycemic control and diabetes-related outcomes represent primary targets.

### 3.3. Overview of Systemic Metabolic Effects

In large-scale clinical trials, semaglutide has demonstrated substantial benefits beyond glycemic reduction, including significant and sustained weight loss, preferential reductions in visceral adiposity, and improvements in cardiometabolic risk profiles [30,31,32]. Notably, these effects often exceed what would be expected from weight loss alone, suggesting the involvement of adipose tissue-specific and systemic metabolic mechanisms.

Emerging meta-analyses and mechanistic investigations further indicate that semaglutide reduces hepatic steatosis, enhances peripheral insulin sensitivity, attenuates adipose tissue inflammation, and modulates lipid metabolism in a pleiotropic manner [31,32]. Collectively, these findings support the concept that adipose tissue represents a central node through which semaglutide integrates metabolic, inflammatory, and endocrine signals, extending its therapeutic impact beyond glucose lowering.

## 4. Effects of Semaglutide on Adipose Tissue Biology in Type 2 Diabetes

### 4.1. Depot-Specific Effects of Semaglutide on Adipose Tissue

**Figure 3:**
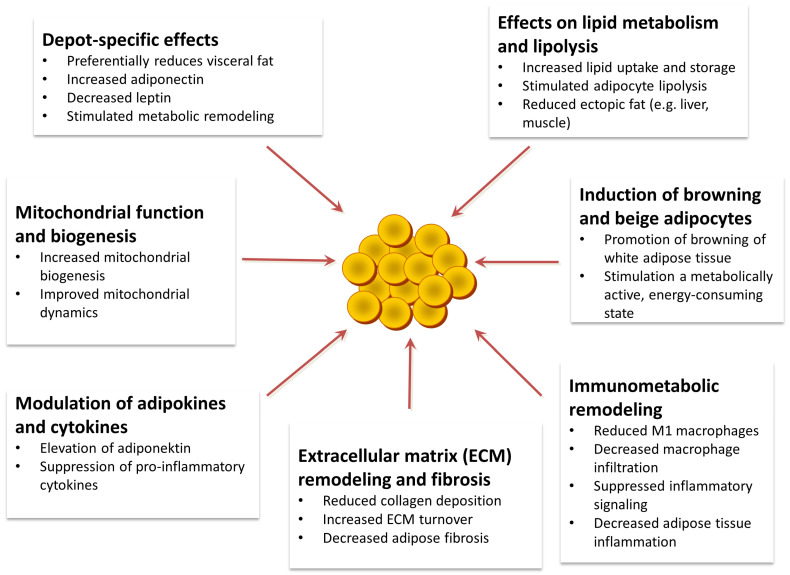
Integrated conceptual overview of adipose tissue remodeling processes associated with semaglutide treatment in type 2 diabetes mellitus.

To enhance clarity and avoid repetition in the presentation of mechanistic evidence, Figure 3 and Table 1 are intentionally designed to serve complementary roles. Figure 3 provides an integrative conceptual overview of semaglutide-associated adipose tissue remodeling, highlighting the interconnection between lipid handling, mitochondrial adaptations, immune modulation, and extracellular matrix dynamics. In contrast, Table 1 serves as an evidence-oriented reference, summarizing key molecular mediators, adipose depot and cell-type specificity, and the relative strength of supporting preclinical versus human data. Together, these elements allow rapid conceptual interpretation (Figure 3) while maintaining transparency regarding the level of experimental and clinical support (Table 1).

Semaglutide exerts significant effects on adipocyte lipid metabolism that extend beyond its indirect impact mediated by weight loss and reduced energy intake [33,34]. Accumulating evidence suggests that semaglutide modulates adipocyte lipid handling through both central and peripheral mechanisms, thereby influencing lipolysis, fatty acid flux, and adipose tissue metabolic flexibility [35].

In preclinical models of obesity and T2DM, semaglutide treatment has been shown to reduce basal and stimulated lipolysis, leading to decreased circulating FFAs levels and attenuation of lipid spillover to peripheral organs [36,37]. This effect appears to be mediated, at least in part, by improved insulin sensitivity within adipocytes, resulting in enhanced suppression of hormone-sensitive lipase activity under insulin-stimulated conditions [37]. These adaptations may contribute to reduced ectopic lipid deposition and improved systemic insulin sensitivity [38].

In addition to regulating lipolysis, semaglutide influences adipocyte lipid storage and turnover. Experimental data indicate that semaglutide promotes a shift toward more efficient triglyceride storage and reduced lipotoxic stress, potentially through improved adipocyte differentiation and lipid droplet remodeling [39]. Such changes may increase the lipid-buffering capacity of adipose tissue, limiting the deleterious metabolic consequences of excess nutrient availability [40].

Clinical studies provide complementary evidence supporting these mechanisms. Treatment with semaglutide has been associated with preferential reductions in visceral adipose tissue mass, improvements in body fat distribution, and decreases in circulating lipid intermediates linked to insulin resistance [30,31]. Importantly, these effects cannot be fully explained by overall weight loss, suggesting a role for adipose tissue-specific metabolic remodeling [32,41].

Within the integrated framework depicted in Figure 3, modulation of adipocyte lipid metabolism and lipolysis represents an upstream mechanistic axis linking semaglutide treatment to downstream improvements in ectopic lipid deposition and systemic insulin sensitivity. Table 1 further details the principal lipid-handling mediators involved in this process (including regulators of lipolysis, fatty acid oxidation, and lipid storage), their depot specificity (visceral versus subcutaneous adipose tissue), and the current strength of evidence supporting each pathway (Figure 3; Table 1).

Taken together, available data indicate that semaglutide improves adipocyte lipid metabolism by enhancing insulin-mediated control of lipolysis, reducing pathological FFAs release, and restoring adipose tissue’s capacity to safely store excess lipids [36,37,38]. These adipocyte-centered effects likely represent a key mechanism through which semaglutide alleviates lipotoxicity and contributes to metabolic improvements beyond glycemic control.

**Table 1:** Evidence-based summary of adipose tissue-centered mechanisms related to semaglutide used in type 2 diabetes mellitus. The table lists the most important molecular mediators and pathways, the main fat stores or cell types, the main effects on adipose tissue, and the relative strength of the evidence (preclinical vs. human studies). This table serves as a detailed reference supplement to the conceptual overview presented in Figure 3.

Mechanistic Domain	Key Molecular Mediators/Pathways	Adipose Depot/Cell Type	Principal Adipose-Related Effect	Evidence Type	Strength of Evidence	References
Depot-specific adipose effects	Preferential reduction in visceral fat; adipokine shift (adiponectin levels increase, leptin levels decrease)	Visceral vs. subcutaneous adipose tissue (SAT); systemic biomarkers	Reduction in visceral adiposity and improvement of adipose endocrine function	Randomized clinical trials; real-world prospective studies	Strong	[8,9,35,36]
Lipid metabolism and lipolysis	Remodeling of lipid-handling proteins (CD36, LPL, FABPs, PLIN2); altered lipid flux	White adipose tissue (predominantly VAT); adipocytes	Reduced lipid uptake and storage programs; improved lipid handling	Preclinical (proteomic analysis in obese mice)	Moderate	[38]
Adipose–liver crosstalk and ectopic fat	Adipocyte-driven hepatic insulin resistance; suppression of hepatic lipogenesis and steatosis	Adipocyte–hepatocyte axis; liver	Reduction in ectopic liver fat and improvement of hepatic metabolic phenotype	Translational (human iPSC-based MPS), preclinical, and clinical MASLD studies	Moderate–Strong	[39,42,43]
Mitochondrial function and biogenesis	AMPK–SIRT1–PGC-1α signaling; oxidative gene programs	Subcutaneous and visceral adipocytes; skeletal muscle	Enhanced mitochondrial biogenesis and oxidative capacity	Preclinical studies and integrative reviews	Moderate	[27,44,45]
Induction of browning and beige adipocytes	UCP1, PRDM16, SIRT1-dependent thermogenic pathways	Predominantly subcutaneous adipose tissue; beige/BAT lineage	Promotion of beige adipocyte programming and thermogenesis	Preclinical semaglutide studies in obese mice; mechanistic reviews	Moderate	[11,13,37,46]
Adipokine and cytokine modulation	Decreased TNF-α, IL-6, CRP; increased insulin-sensitizing adipokines	Adipose tissue secretome; systemic circulation	Attenuation of chronic low-grade inflammation	Meta-analyses and mechanistic reviews	Strong	[22,47,48,49]
Immunometabolic remodeling	Reduced macrophage infiltration; STAT3-mediated M2 polarization; suppression of NF-κB/JNK signaling	Adipose tissue macrophages (ATM)	Shift toward an anti-inflammatory immune milieu	Preclinical models and in vitro human macrophage studies	Moderate	[12,49,50,51]
Extracellular matrix remodeling and fibrosis	Collagen VI (COL6A3), LOX, MMP/TIMP balance; peri-adipocyte ECM remodeling	Fibrotic adipose microenvironment	Reduced fibrotic constraints and improved adipose tissue expandability	Mechanistic adipose fibrosis literature; comparative pharmacotherapy studies	Limited–Moderate	[18,52,53,54,55]
Adipose tissue cellular composition	Distinct remodeling of white adipose tissue cellular landscape vs. bariatric surgery	Mouse white adipose tissue	Qualitative reshaping of adipose cellular ecosystem	Preclinical tissue-level studies	Limited	[11,14]

ATM, adipose tissue macrophage; BAT, brown adipose tissue; CRP, C-reactive protein; ECM, extracellular matrix; iPSC-MPS, induced pluripotent stem cell-based microphysiological system; MASLD, metabolic dysfunction-associated steatotic liver disease; SAT, subcutaneous adipose tissue; VAT, visceral adipose tissue.

The schematic diagram summarizes the main mechanistic areas, including adipocyte lipid handling and fatty acid flux, mitochondrial biogenesis and oxidative capacity, beige adipocyte programming, immunometabolic remodeling, adipokine/cytokine regulation, and extracellular matrix (ECM) dynamics, and illustrates their functional relationships. Specific molecular mediators, depot/cell-type specificity, and strength of evidence are summarized separately in Table 1.

### 4.2. Effects on Lipid Metabolism and Lipolysis

T2DM is marked by insulin resistance and dysregulated adipose lipolysis, leading to elevated FFAs and ectopic fat deposition. Importantly, adipocytes express GLP-1R, allowing GLP-1 RA to act directly on fat tissue [39]. In obese mice models, semaglutide significantly reduced adiposity and adipocyte size while activating “browning” of white fat [11]. Martins et al. observed that semaglutide treatment increased UCP1 labeling, promoted thermogenic gene expression necessary for maintaining the browning phenotype: beta-3 adrenergic receptor (+520%), PR domain-containing 16 (+90%), and UCP1 (+110%) while reducing inflammatory markers [11].

Proteomic analysis of epididymal fat from high-fat-fed mice showed that semaglutide downregulated multiple lipid-handling proteins—including CD36, FABP5, ACSL, PLIN2, ANGPTL4, LPL, MGLL, AQP7 and PDK4—which is consistent with decreased lipid uptake/storage [38]. Together, these data indicate that semaglutide shifts adipose metabolism toward enhanced lipid mobilization and oxidation. Notably, the direction of lipolytic modulation may depend on the metabolic context and experimental setting: in insulin-resistant states, semaglutide can improve insulin-mediated suppression of pathological basal lipolysis, while in mechanistic in vitro systems it may enhance stimulated lipid mobilization coupled to increased fatty acid oxidation and downstream metabolic remodeling.

Semaglutide stimulates lipolysis in adipocytes [39]. In a human iPSC-derived adipose–liver microphysiological model, semaglutide markedly increased adipocyte lipolysis: treated adipocyte cultures showed elevated hormone-sensitive lipase (HSL) expression and released more fatty acids into the media [39]. This was accompanied by higher adiponectin gene expression and secretion (increasing insulin-sensitizing adipokine levels) and a sharp reduction in inflammatory cytokine TNFα secretion [12,40]. Parallel gene analysis in treated white adipocytes showed upregulation of GLUT4 and lipolytic genes (e.g., HSL) [40], suggesting that semaglutide promotes the breakdown of stored triglycerides. Consistent with these findings, semaglutide upregulates key β-oxidation regulators (e.g., peroxisome proliferator-activated receptor α (PPARα), carnitine palmitoyltransferase 1A (CPT1A), peroxisome proliferator-activated receptor-gamma co-activator 1-α (PGC-1α) in adipose and liver of obese mice [38,41]. Overall, semaglutide activates adipocyte lipolysis and mitochondrial fat burning, in part via adipose GLP-1R signaling.

By increasing peripheral lipid clearance and oxidation, semaglutide also reduces ectopic fat. In obese and T2DM mice, the semaglutide decreased liver and muscle lipid accumulation [27,42]. In the human microphysiology model, semaglutide-treated adipocyte media (high in fatty acids from enhanced lipolysis) led to strikingly lower hepatocyte lipid content [39]. This coincided with downregulation of hepatic lipogenesis genes (ACCα, ACCβ, FASN) and upregulation of PPARα (fatty acid oxidation), suggesting semaglutide drives the liver away from storing fat and toward burning it [39].

Clinically, semaglutide 1 mg once weekly robustly lowers liver steatosis: one 16-week trial in obese men (n = 16) showed ~5% body weight loss but a 33% decrease in magnetic resonance imaging (MRI)-measured liver fat, independent of weight loss [43]. Lipidomic analysis from this study revealed reduced palmitate and palmitoleate fractions in very low-density lipoprotein (VLDL)-triglycerides after semaglutide, indicating suppressed hepatic de novo lipogenesis [43]. Although these participants were primarily characterized by obesity and metabolic dysfunction-associated steatotic liver disease (MASLD) rather than T2DM-specific endpoints, these results provide translational support for adipose tissue-liver metabolic improvements beyond glucose reduction.

In circulation, semaglutide improves the atherogenic lipid profile. Meta-analysis of trials in overweight/obese subjects found that once-weekly semaglutide (2.4 mg) significantly reduced fasting total cholesterol, low-density lipoprotein cholesterol (LDL-C), VLDL-cholesterol, and triglycerides (TG), while modestly raising high-density lipoprotein (HDL) cholesterol [56]. Pooled data showed a mean fall in TG of ~15 mg/dL and in LDL-C of ~6 mg/dL at 68 weeks [56]. Consistent with this research, a human hepatic fat study reported that semaglutide (unlike a diet control) significantly lowered plasma TG and VLDL-C levels, even though fasting non-esterified fatty acids (NEFA) and total cholesterol were unchanged [56]. Thus, semaglutide appears to reduce triglyceride-rich lipoproteins and shift LDL/HDL subfractions to a less atherogenic profile, contributing to its cardiometabolic benefit in T2DM with obesity.

High-throughput profiling has begun to map semaglutide’s effects on lipid networks. Proteomics of adipose tissue (mice) and serum (humans) reveal broad regulation of lipid-handling pathways. Zhu et al. found that approximately 640 adipose proteins were altered by semaglutide in obese mice [38]. Notably, many downregulated proteins were tied to fatty acid uptake and storage (e.g., CD36, LPL, FABPs), while pathways for energy expenditure were enriched [38]. In human trials, large-scale serum proteomics also showed semaglutide-induced shifts in proteins related to lipid metabolism, inflammation, and cardiovascular risk [57]. Taken together, these data suggest semaglutide lowers circulating lipotoxic factors (e.g., leptin, inflammatory mediators) and upregulates beneficial adipokines.

Metabolomic analyses likewise reflect enhanced fat oxidation. For instance, VLDL-triglyceride profiling in obese patients revealed that semaglutide treatment reduced saturated fatty acid content (palmitate and palmitoleate) in circulating TG, consistent with suppressed hepatic lipogenesis [43]. Although comprehensive metabolomic data are limited, these changes align with observed shifts in plasma lipids. In summary, semaglutide reprograms lipid metabolism in T2DM by promoting adipocyte lipolysis and fatty acid oxidation, reducing lipid storage in liver and muscle, and improving systemic lipid profiles [39,40,41,42,43,56,57]. These effects are supported by coordinated changes at the proteomic and metabolomic level in both preclinical models and clinical studies.

### 4.3. Mitochondrial Function and Biogenesis

In adipocytes, semaglutide upregulates key transcriptional regulators of mitochondrial biogenesis—PGC-1α, nuclear respiratory factor1 (NRF1), and mitochondrial transcription factor (TFAM)—driving the formation of new mitochondria [11]. In diet-induced obese mice, this corresponds to large increases (∼110–260%) in these factors, along with higher UCP1 expression and “browning” of white fat [11]. The result is more abundant and more oxidative mitochondria in adipose tissue. Semaglutide also acts directly on muscle GLP-1 receptors: in skeletal muscle, it raises PGC-1α and related antioxidant genes, suggesting a systemic boost in mitochondrial content [10]. Overall, semaglutide-treated tissues show higher mitochondrial density and oxidative enzyme expression, increasing their ATP production capacity [10]. However, most human evidence in this domain is indirect and largely derived from systemic endpoints measured in clinical trials (e.g., changes in body composition or circulating metabolic markers) rather than from direct mitochondrial phenotyping of human adipose tissue in either T2DM or obesity cohorts.

In addition to increasing biogenesis, semaglutide promotes healthier mitochondrial dynamics. It has been shown to prevent excessive fission and support fusion (e.g., maintaining optic atrophy 1/mitofusins2 (OPA1/Mfn2) levels) under metabolic stress [57]. In pressure-overload hearts, semaglutide treatment preserved normal tubular mitochondrial networks rather than allowing fragmentation [58]. A shift toward a fused network enhances respiratory efficiency. Consistent with this, GLP-1R agonists (including semaglutide) increase mitochondrial size/number and thermogenic gene expression in fat and muscle, primarily in preclinical experimental models. However, direct evidence demonstrating comparable mitochondrial network remodeling in human adipose tissue—either in T2DM or in non-diabetic obesity cohorts—remains limited [44]. In practice, this means adipocytes and muscle cells develop larger, more interconnected mitochondria with higher UCP1 and other heat-generating proteins. These changes favor greater fatty acid oxidation and thermogenesis.

These mitochondrial adaptations translate into improved metabolic flexibility and insulin sensitivity. By boosting mitochondrial number and function, semaglutide allows cells to burn lipids more effectively. In semaglutide-treated animals, cardiomyocytes showed increased pyruvate entry into the tricarboxylic acid cycle (TCA cycle), complete glucose oxidation, and higher fatty acid oxidation rates [58]. Similarly in adipose, elevated mitochondrial content and UCP1 expression accelerate lipid turnover and heat production [11]. Overall, these effects—greater oxidative capacity, fused mitochondrial networks, and thermogenesis—increase metabolic flexibility (the ability to switch between fuels) and counteract insulin-resistant phenotypes. Nevertheless, confirmation in human T2DM versus obesity cohorts requires dedicated adipose tissue studies.

### 4.4. Induction of Browning and Beige Adipocytes

Semaglutide has been shown to promote the browning of white adipose tissue (WAT), particularly within subcutaneous depots. Browning refers to the transdifferentiation or de novo differentiation of white adipocytes into beige adipocytes, a distinct cell type characterized by multilocular lipid droplets, increased mitochondrial density, and expression of thermogenic genes such as UCP1 [11]. This process enhances energy expenditure and is associated with improved metabolic flexibility and glycemic control.

In a seminal study by Martins et al., semaglutide treatment in high-fat diet-induced obese mice led to significant upregulation of UCP1, PR domain containing 16 (Prdm16), and β3-adrenergic receptor (β3-ar) expression in subcutaneous adipose tissue [11]. Histological analysis revealed an increased number of multilocular adipocytes, consistent with beige cell morphology, along with a significant reduction in mean adipocyte diameter. These morphological and molecular adaptations were accompanied by improved insulin sensitivity and downregulation of inflammatory markers such as TNF-α and IL-6 [11].

Additional transcriptomic and proteomic evidence further supports that GLP-1 RAs enhance mitochondrial biogenesis and fatty acid oxidation in WAT, both considered key hallmarks of beige adipocyte programming. Upregulation of oxidative genes such as PGC-1α and CPT1A has been observed in white adipose depots following GLP-1 RA treatment in preclinical models [45]. These changes reflect a shift in adipose tissue from a storage-oriented phenotype toward a metabolically active, energy-dissipating state.

Supporting evidence from in vitro human cell models also suggests that GLP-1 RA may promote a beige/thermogenic program in human adipocytes. In human SGBS adipocytes cultured under adipogenic conditions, long-term treatment with the GLP-1 RA liraglutide increased mitochondrial respiration and upregulated UCP1 together with markers of mitochondrial biogenesis, indicating enhanced thermogenic capacity and partial browning of white adipocytes [59]. Although semaglutide has not yet been systematically evaluated in equivalent human adipocyte or mesenchymal stem cell models, these findings support the concept that GLP-1RA signaling can directly favor a beige-like phenotype in human adipose cells. Therefore, current clinical interpretations rely largely on indirect markers of metabolic improvement rather than direct confirmation of beige adipocyte recruitment in vivo.

The induction of beige adipocytes by semaglutide and related GLP-1 RAs likely contributes to their metabolic benefits. Beige adipocytes exhibit elevated basal and inducible thermogenesis, increase whole-body energy expenditure, and may facilitate weight loss beyond appetite suppression. Moreover, the shift toward an oxidative adipocyte phenotype mitigates lipotoxic stress and improves systemic insulin sensitivity [46]. Altogether, these findings position adipose tissue browning as a putative mechanistic axis through which semaglutide may exert its pleiotropic actions in metabolic disease, warranting further validation in studies focusing on human adipose tissue.

### 4.5. Modulation of Adipokines and Cytokines

Semaglutide modulates the adipose tissue secretome by increasing circulating adiponectin while suppressing pro-inflammatory cytokines such as TNF-α and IL-6. This cytokine shift may enhance systemic insulin sensitivity and attenuate obesity-associated low-grade inflammation. Proteomic and clinical data support that semaglutide regulates a broad set of proteins beyond those linked strictly to weight loss or glycemic control, including downregulation of inflammatory pathways and proteins associated with lipogenesis and adipose tissue dysfunction [57].

In large randomized trials and meta-analyses, semaglutide therapy has been associated with reductions in systemic inflammatory markers—notably C-reactive protein (CRP), and in many studies also IL-6 and TNF-α—supporting an overall anti-inflammatory effect in people with T2DM or obesity [47,48]. It is important to note that although these systemic anti-inflammatory effects are observed in both populations, the baseline inflammatory burden, semaglutide dose exposure, and degree of weight loss may differ between patients with T2DM and individuals treated primarily for obesity, which should be taken into account when interpreting mechanisms related to adipose tissue.

Preclinical evidence further supports direct immunomodulatory effects of GLP-1 RA in murine models; semaglutide reduced systemic cytokines (e.g., TNF-α, IL-6), decreased immune cell recruitment into tissues, and downregulated chemokines and pro-inflammatory signaling pathways, consistent with improved adipose tissue immune environment [49]. However, these mechanistic findings are primarily derived from experimental models, and their direct confirmation in human adipose tissue remains restricted.

Taken together, these data suggest that semaglutide reprograms adipose tissue secretory function—shifting it toward a more anti-inflammatory, insulin-sensitizing adipokine/cytokine profile. This may represent a key mechanistic axis, complementing its effects on adiposity and energy balance, and contributing to improved metabolic and cardiometabolic outcomes in T2DM and obesity.

### 4.6. Immunometabolic Remodeling

Beyond its established effects on adipocyte biology, semaglutide may exert immunometabolic regulatory effects within adipose tissue. Chronic low-grade inflammation in obesity and T2DM is largely sustained by an imbalance in adipose-resident immune cell populations—notably an excess of pro-inflammatory M1 macrophages, infiltrating macrophages, and other innate immune effectors—which disrupt insulin signaling and promote lipotoxic stress [12,50].

Preclinical data show that GLP-1—and by extension GLP-1R agonists—can modulate macrophage polarization: in vitro treatment of macrophages with GLP-1 reduced M1-specific markers (e.g., Inducible Nitric Oxide Synthase (iNOS), TNF-α, IL-6) and increased M2-associated markers (e.g., IL-10, CD163, CD204) via signaling pathways involving increased signal transducer and activator of transcription 3 (STAT3) activation, thereby promoting an anti-inflammatory M2 phenotype [51].

In obese murine models, GLP-1 receptor activation has been demonstrated to reduce macrophage infiltration into adipose tissue and to suppress inflammatory signaling (Nuclear factor-κB (NF-κB), jun N-terminal kinase (JNK)) in both adipocytes and adipose tissue macrophages (ATM), changes that were associated with improved insulin sensitivity [12].

Recently, comprehensive reviews summarizing the metabolic effects of GLP-1R agonists—including liraglutide and semaglutide—have reported reductions in pro-inflammatory adipokine expression, decreased adipose tissue inflammation, and improvements in systemic insulin sensitivity [27,29].

Thus, semaglutide likely contributes to immunometabolic remodeling of adipose tissue by reducing macrophage-mediated inflammation, shifting the macrophage phenotype toward the M2 (anti-inflammatory) state, and suppressing key inflammatory signaling pathways. These changes may stabilize the adipose tissue microenvironment, support better adipocyte function, and contribute to improved systemic glucose homeostasis.

Collectively, semaglutide should be viewed not merely as an incretin mimetic, but—in the context of obesity/T2DM—potentially as a modulator of adipose-tissue immunobiology, aligning metabolic and immune pathways toward a lower-inflammatory, more insulin-sensitive state. However, it must be noted that much of the mechanistic evidence comes from preclinical or animal studies; confirmation in human adipose tissue remains limited, and further research is needed to firmly establish this immunometabolic axis as a key mediator of semaglutide’s extra-glycemic benefits.

### 4.7. Extracellular Matrix Remodeling and Fibrosis

Adipose tissue fibrosis represents a critical barrier to metabolic flexibility in T2DM, arising from dysregulated ECM turnover, excessive collagen deposition, and stromal stiffening [52]. These structural alterations impair adipocyte expandability, exacerbate hypoxia, promote inflammatory signaling, and restrict insulin-stimulated glucose uptake, thereby reinforcing insulin resistance and adipose tissue dysfunction [52,60]. The accumulation of fibrillar collagens—particularly collagen type VI alpha 3 chain (COL6A3) and collagen I—is a hallmark of metabolically impaired adipose depots, and their crosslinking by lysyl oxidase (LOX) further increases ECM rigidity and mechanical stress on adipocytes [53]. The resultant mechano-transduction activates Yes-associated protein (YAP) and transcriptional co-activator with PDZ-binding motif (TAZ) pathways, which inhibit adipogenesis and favor a pro-inflammatory, lipotoxic microenvironment [54].

Emerging evidence indicates that semaglutide modulates adipose ECM structure by attenuating fibrogenic signaling and enhancing ECM turnover [61]. In high-fat diet-induced obese murine models, semaglutide reduced expression of fibrotic genes including collagen type I alpha 1 (COL1A1), COL6A3, and LOX, an effect partly attributed to activation of AMP-activated protein kinase (AMPK) and downstream SIRT1/PGC-1α pathways, which repress profibrotic transcriptional regulators [11,14]. In a recent single-cell transcriptomic study, semaglutide induced transcriptional changes in stromal and immune cell subsets of epididymal white adipose tissue, including significant downregulation of fibrotic and inflammatory genes and pathways, along with upregulation of mitochondrial and oxidative metabolism markers [14]. Concurrently, semaglutide normalized the balance between matrix metalloproteinases (MMPs) and their inhibitors (TIMPs), leading to increased MMP-2 and MMP-9 activity and reduced TIMP-1 levels, changes indicative of enhanced ECM remodeling and decreased matrix accumulation [55].

These molecular adaptations are accompanied by reduced adipocyte size, improved mitochondrial biogenesis, and a shift toward oxidative, beige-like adipocyte phenotypes, suggesting that ECM remodeling and metabolic reprogramming occur concurrently rather than independently [45,61]. Furthermore, semaglutide’s antifibrotic effects appear to intersect with immunometabolic signaling. By reducing ECM-driven macrophage recruitment and suppressing NF-κB and JNK pathway activation, semaglutide may indirectly promote anti-inflammatory M2 macrophage polarization and decrease the release of damage-associated molecular patterns (DAMPs) from fibrotic adipose tissue [12,51]. Collectively, these processes contribute to an adipose microenvironment that supports insulin sensitivity, preserves adipogenic capacity, and enhances whole-body metabolic flexibility.

Taken together, these findings suggest that semaglutide exerts direct and indirect antifibrotic effects within adipose tissue through activation of AMPK–SIRT1–PGC-1α signaling and restoration of ECM proteolytic balance. Given the established association between adipose fibrosis, visceral adiposity, and cardiometabolic risk, the capacity of semaglutide to remodel adipose ECM may represent a key molecular mechanism contributing to its metabolic benefits beyond glycemic control. However, as most mechanistic insights derive from rodent models, further studies are warranted to determine whether antifibrotic responses predict therapeutic efficacy or represent biomarkers of semaglutide responsiveness in human T2DM.

## 5. Conclusions and Perspectives

Accumulating evidence indicates that semaglutide represents a new generation of GLP-1 RAs whose therapeutic effects may extend substantially beyond glycemic control, positioning adipose tissue as a central target organ in T2DM. By orchestrating coordinated changes in adipocyte lipid handling, mitochondrial biogenesis, beige adipocyte programming, adipokine secretion, immune cell composition, and extracellular matrix (ECM) dynamics, semaglutide can induce a profound functional remodeling of adipose tissue toward a more oxidative, insulin-sensitive, and metabolically flexible phenotype [11,14,62,63,64]. It is important to note, however, that much of the mechanistic knowledge underlying these processes comes from experimental obesity/T2DM models, while evidence in humans remains more limited and often based on systemic endpoints.

This adipose tissue remodeling appears to serve as a mechanistic hub linking semaglutide-induced weight loss to improvements in systemic insulin sensitivity, reduction in ectopic lipid accumulation, attenuation of chronic low-grade inflammation, and protection against cardiometabolic complications [65,66]. Importantly, several of these effects—such as reductions in visceral adiposity, normalization of adipokine profiles, and suppression of adipose inflammation—cannot be fully explained by weight loss alone, underscoring the presence of direct and indirect adipose-specific mechanisms [47,67,68]. However, the extent and underlying causes of these effects may differ between patients with T2DM and those treated primarily for obesity, given differences in baseline metabolic status, comorbidities, and typical dosing regimens.

From a translational perspective, these findings may support a conceptual shift from glucose-centric to organ-centric metabolic therapy. Adipose tissue-focused biomarkers, including depot-specific imaging, circulating adipokines, inflammatory mediators, and ECM-related factors, may emerge as valuable tools for predicting therapeutic response and stratifying patients most likely to benefit from semaglutide treatment [69,70]. Integration of multi-omics approaches—such as single-cell transcriptomics, proteomics, and metabolomics—may be critical for disentangling cell-type-specific effects of semaglutide within adipose depots and for identifying novel molecular signatures of treatment responsiveness [14,57].

Based on current clinical evidence, combination therapeutic strategies targeting complementary metabolic pathways appear particularly promising. In this context, co-administration of sodium–glucose cotransporter 2 (SGLT2) inhibitors with GLP-1 RAs, including semaglutide, has demonstrated favorable safety and efficacy profiles, along with additive cardiometabolic and vascular benefits in patients with T2DM [71,72]. These findings support further investigation of rational combination approaches to enhance therapeutic outcomes by concurrently addressing multiple aspects of metabolic dysfunction.

However, despite compelling preclinical data, human adipose tissue-centric mechanistic studies remain limited. Longitudinal clinical trials incorporating adipose biopsies, advanced imaging, and immunometabolic profiling are essential to validate these mechanisms in patients with T2DM. It is important to note that adipose tissue biology exhibits significant sex dimorphism, with well-described sex- and depot-specific differences in adipose tissue cellular composition, endocrine function, and inflammatory regulation [73]. However, most of the experimental studies summarized in this review involved male animals or did not systematically report sex-specific outcomes. Moreover, although sex-specific adipose tissue biology has been extensively characterized, semaglutide-focused mechanistic studies—particularly in humans—rarely provide sex-stratified, depot-resolved molecular profiling, limiting conclusions regarding potential sex-dependent adipose tissue responses to treatment.

In conclusion, semaglutide may exemplify a paradigm shift in metabolic disease management as it can target adipose tissue as a dynamic regulator of systemic metabolic health. Elucidating the cellular and molecular networks underlying its potential adipose-directed actions may not only refine its clinical use but also inform the development of next-generation incretin-based and adipose-targeted therapies for T2DM and cardiometabolic disease.

## Data Availability

No new data were created or analyzed in this study. Data sharing is not applicable to this article.

## Abbreviations

The following abbreviations are used in this manuscript:

T2DM	Type 2 diabetes mellitus
ECM	Extracellular matrix
GLP-1RAs	Glucagon-like peptide-1 receptor agonists
VAT	Visceral adipose tissue
IL-6	Interleukin-6
TNF-α	Tumor necrosis factor-α
CCL-2	C-C motif chemokine ligand 2
IRS-1	Insulin receptor substrate 1
GLUT4	Glucose transporter type-4
FFAs	Free fatty acids
GLP1-RA	Peptide-1 receptor agonist
CNS	Central nervous system
GLP1-R	GLP-1 receptor
DPP-4	Dipeptidyl peptidase-4
HSL	Hormone-sensitive lipase
PPARα	Peroxisome proliferator-activated receptor α
CPT1A	Carnitine palmitoyltransferase 1A
PGC-1α	Peroxisome proliferator-activated receptor-gamma co-activator 1-α
MRI	Magnetic resonance imaging
VLDL	Very low-density lipoprotein
MASLD	Metabolic dysfunction-associated steatotic liver disease
LDL	Low-density lipoprotein
TG	Triglycerides
HDL	High-density lipoprotein
NEFA	Non-esterified fatty acids
NRF1	Nuclear respiratory factor1
TFAM	Mitochondrial transcription factor
OPA1/Mfn2	Optic atrophy 1/mitofusins2
TCA cycle	Tricarboxylic acid cycle
WAT	White adipose tissue
Prdm16	PR domain containing 16
β3-ar	β3-adrenergic receptor
iNOS	Nitric Oxide Synthase
STAT3	Signal transducer and activator of transcription 3
NF-κB	Nuclear factor-κB
JNK	Jun N-terminal kinase
ATM	Adipose tissue macrophages
COL6A3	Collagen type VI alpha 3 chain
LOX	Lysyl oxidase (LOX)
YAP	Yes-associated protein
TAZ	Transcriptional co-activator with PDZ-binding motif
COL1A1	Collagen type I alpha 1
AMPK	AMP-activated protein kinase
MMPs	Matrix metalloproteinases
DAMPs	Damage-associated molecular patterns
SGLT2	Sodium–glucose cotransporter 2
